# Distribution of Cytoglobin in the Mouse Brain

**DOI:** 10.3389/fnana.2016.00047

**Published:** 2016-04-27

**Authors:** Stefan Reuss, Sylvia Wystub, Ursula Disque-Kaiser, Thomas Hankeln, Thorsten Burmester

**Affiliations:** ^1^Department of Nuclear Medicine, University Medical Center, Johannes Gutenberg-UniversityMainz, Germany; ^2^Institute of Molecular Genetics, Johannes Gutenberg-UniversityMainz, Germany; ^3^Department of Anatomy and Cell Biology, University Medical Center, Johannes Gutenberg-UniversityMainz, Germany; ^4^Institute of Zoology and Zoological Museum, University of HamburgHamburg, Germany

**Keywords:** cytoglobin, globin, immunofluorescence, neuroanatomy, mouse brain

## Abstract

Cytoglobin (Cygb) is a vertebrate globin with so far poorly defined function. It is expressed in the fibroblast cell-lineage but has also been found in neurons. Here we provide, using immunohistochemistry, a detailed study on the distribution of Cygb in the mouse brain. While Cygb is a cytoplasmic protein in active cells of the supportive tissue, in neurons it is located in the cytoplasm and the nucleus. We found the expression of Cygb in all brain regions, although only a fraction of the neurons was Cygb-positive. Signals were of different intensity ranging from faint to very intense. Telencephalic neurons in all laminae of the cerebral cortex (CCo), in the olfactory bulb (in particular periglomerular cells), in the hippocampal formation (strongly stained pyramidal cells with long processes), basal ganglia (scattered multipolar neurons in the dorsal striatum, dorsal and ventral pallidum (VP)), and in the amygdala (neurons with unlabeled processes) were labeled by the antibody. In the diencephalon, we observed Cygb-positive neurons of moderate intensity in various nuclei of the dorsal thalamus, in the hypothalamus, metathalamus (geniculate nuclei), epithalamus with strong labeling of habenular nucleus neurons and no labeling of pineal cells, and in the ventral thalamus. Tegmental neurons stood out by strongly stained somata with long processes in, e.g., the laterodorsal nucleus. In the tectum, faintly labeled neurons and fibers were detected in the superior colliculus (SC). The cerebellum exhibited unlabeled Purkinje-neurons but signs of strong afferent cortical innervation. Neurons in the gray matter of the spinal cord showed moderate immunofluorescence. Peripheral ganglia were not labeled by the antibody. The Meynert-fascicle and the olfactory and optic nerves/tracts were the only Cygb-immunoreactive (Cygb-IR) fiber systems. Notably, we found a remarkable level of colocalization of Cygb and neuronal nitric oxide (NO)-synthase in neurons, which supports a functional association.

## Introduction

The globin family comprises small porphyrin-containing proteins that reversibly bind O_2_ by means of an iron (Fe^2+^) ion of the heme prosthetic group (Weber and Vinogradov, 2001; Wittenberg and Wittenberg, 2003; Burmester and Hankeln, 2014). Apart from the well-known respiratory proteins hemoglobin (Hb) and myoglobin (Mb), the vertebrate globin family has six other members: Neuroglobin (Ngb; Burmester et al., 2000), cytoglobin (Cygb; Kawada et al., 2001; Burmester et al., 2002; Trent and Hargrove, 2002), globin E (GbE; Kugelstadt et al., 2004; Blank et al., 2011), globin X (GbX; Roesner et al., 2005; Blank and Burmester, 2012), globin Y (GbY; Fuchs et al., 2006), and androglobin (Adgb; Hoogewijs et al., 2012). The vertebrate globins have an evolutionarily complex history and may serve different functions which are, however, often only poorly defined (Burmester and Hankeln, 2014). While GbE, GbX and GbY apparently have been lost in mammals and few other vertebrate taxa (Burmester and Hankeln, 2014), Hb, Mb, Ngb, Cygb and Adgb were found in most jawed vertebrates (Hoogewijs et al., 2012; Schwarze and Burmester, 2013).

Cygb is an early offshoot of the vertebrate globin family that emerged in the vertebrate stem-lineage before the typical respiratory globins, Hb and Mb, evolved (Schwarze et al., 2014; Rohlfing et al., 2016). Cygb was first identified in hepatic stellate cells, where it showed enhanced expression in fibrosis, and was thus named “Stellate cell activation-associated protein” (STAP; Kawada et al., 2001; Asahina et al., 2002). However, this heme-protein actually is expressed in a broad range of tissues and has, therefore, received the official designation “Cygb” (Burmester et al., 2002; Schmidt et al., 2004). More detailed studies indicated an exclusively cytoplasmic localization of Cygb in fibroblasts and related cell types such as chondroblasts and osteoblasts (Nakatani et al., 2004; Schmidt et al., 2004).

The molecular function of Cygb is still elusive. The cellular distribution of Cygb does not correlate with O_2_-consumption, rendering a myoglobin-like respiratory role rather implausible (Hankeln et al., 2005; Schmidt et al., 2005). Cygb may decompose reactive oxygen species (ROS) as it transfers ROS-protection to cultured cells (Xu et al., 2006; Li et al., 2007; Hodges et al., 2008; Fang et al., 2011; Singh et al., 2014). Other hypotheses suggest that Cygb supplies molecular O_2_ to enzymatic processes such as collagen synthesis (Schmidt et al., 2004) or the production of nitric oxide (NO; Hankeln et al., 2005) or may be involved in a lipid-based signaling process (Reeder et al., 2011). There is also evidence that Cygb is involved in the formation of cancer (reviewed by Oleksiewicz et al., 2011; Thuy Le et al., 2016).

Additionally to the broad expression in fibroblast cell lineage, Cygb was detected in some neuronal subpopulations in distinct brain sites including the retina (Schmidt et al., 2004, 2005). In contrast to the exclusively cytoplasmic localization in the supportive tissues, immunohistochemical studies revealed the localization of Cygb concurrently in both, the cytoplasm as well as the nucleus of neurons. This observation strongly indicates a different or additional role of Cygb in neuronal tissues. This interpretation is tentatively supported by the existence of two paralogous Cygb-genes in fish (Fuchs et al., 2005), which display distinct kinetic features (Corti et al., 2016).

Cygb-positive neurons were found in mouse (Schmidt et al., 2004, 2005; Hundahl et al., 2010), the blind mole rat Spalax (Avivi et al., 2010), rat and man (Hundahl et al., 2013). In these studies, the expression of this globin was only cursorily studied in restricted brain regions or concerning the neurochemical phenotypes of Cygb-neurons. Since a detailed analysis of the distribution of Cygb protein in many regions of the brain was missing, we conducted an immunofluorescence study to provide a comprehensive report on the distribution of Cygb-neurons and the differential strengths of respective immunofluorescence in the mouse brain.

Since a hypothetical Cygb function is to provide oxygen to intracellular enzymatic reactions, the colocalization of Cygb and neuronal nitric oxide-synthase (nNOS), the enzyme responsible for production of the neuronal messenger NO, was proposed (Hankeln et al., 2004, 2005) and recently demonstrated (Avivi et al., 2010; Hundahl et al., 2010), we also tested neurons in selected brain regions for the colocalization of Cygb and nNOS.

## Materials and Methods

Adult mice (Balb/C, five females, five males, 3 months old) used in this study were maintained under constant conditions (light:dark cycle 12:12 h, room temperature (RT) 21 ± 1°C) with food and water *ad libitum*. The protocol was approved by the local Administration District Official Committee and was in accordance with the published European Health Guidelines. All efforts were made to minimize the number of animals and their suffering.

At the middle of the light period, the mice were killed by an ether overdose and immediately perfused transcardially with 50 ml of room-temperatured 0.1 M phosphate-buffered saline (PBS), with 15,000 IU heparin/l added. Perfusion-fixation was carried out using 250 ml of ice-cold 4% paraformaldehyde, 1.37% L-lysine, 0.21% sodium-periodate in PBS (McLean and Nakane, 1974). The right atrium was opened to enable venous outflow. The brain was removed, postfixed for 1 h in the same fixative and stored at 4°C in PBS until further processing.

After cryoprotection in 30% sucrose, the brains were sectioned at 40 μm thickness in the frontal plane on a freezing microtome. Non-specific binding sites were blocked at RT for 1 h with 1% bovine serum albumin (BSA) in PBS. The sections were incubated with rabbit-raised antibodies directed against the amino acid positions 2–16 of the N-terminus (1:1000 in PBS, to which 2% normal donkey serum and 0.2% Triton-X 100 were added (for further antibody details, see Schmidt et al., 2004) overnight. The sections were washed 3 × 10 min in PBS and incubated for 90 min at RT in the dark with the secondary antibody (Cy3-conjugated F(ab′)2-fragments of goat anti-rabbit IgG, 1:300 in PBS, Dianova, Hamburg, Germany). Brain sections from each single animal were incubated simultaneously and under identical conditions so that the fluorescence intensity levels are relative to each other.

Some sections were incubated at RT for 15 min in a 400 nM solution of 4′-6-diamidino-2-phenylindole (DAPI, in PBS, Molecular Probes, Eugene, OR, USA) for nuclear staining. For double-immunostaining experiments, selected sections were incubated in primary Cygb-antibody as described above and simultaneously, in either a polyclonal sheep antibody raised against (nNOS, 1:50, Abcam, Cambridge, UK) or a mouse monoclonal antibody raised against tyrosine hydroxylase (TH, 1:200, Chemicon, Temecula, CA, USA). Both antibodies were used and characterized in our laboratory previously (Reuss et al., 2009a,b). The additional primary antibodies were visualized using Cy2-conjugated F(ab′)2-fragments of anti-sheep or anti-mouse IgG (Dianova) at 1:300 dilution. Sections were washed as described above, mounted on gelatinized glass slides, dried, cleared in xylene and covered.

All sections were analyzed using an Olympus BX51 research microscope equipped with an epifluorescence unit, highly specific single band filter sets allowing the excitation and observation of dyes without overlapping artifacts (Olympus fluorescence mirror cubes, maximal excitation/maximal emission, Cy2: 489/506 nm, Cy3: 552/565 nm). Photomicrographs were taken with a digital color camera. Images were taken using the Analysis Software (Soft Imaging System, Münster, Germany) and when indicated in legends, arranged using the options “multiple image alignment” (MIA) and “extended focal imaging” (EFI). Specimens were analyzed using incubated sections as well as photomicrographs taken from sections. The relative intensity of Cygb-IR neuronal somata (with +++ as the highest levels) in the studied regions are presented in Table 1.

**Table 1:** **Distribution of cytoglobin-immunoreactive (Cygb-IR) neuronal somata in the mouse brain**.

Brain regions	Level^1^	Figure	Additional remarks
**1. Telencephalon**
**1.1 Cerebrum**
**1.1.1 Olfactory bulb**
Olfactory nerve layer	+	1A	Fibers, periglom. neurons
Glomerular layer	++	1A	Glom. axons/dendrites
External plexiform layer	+	1A	Single neurons
Mitral cell layer	+	1A
Internal plexiform layer	+
Granule cell layer	+
Subependymal cell layer	+++		Deep short-axon cells
Anterior olfactory nucleus	+/++	1B,D,E
Accessory olfactory bulb	+
**1.1.2 Cerebral cortex**
Layers I–VI	+/++/+++	1B,C, 2A,B	Single neurons, all area
**1.1.3 Hippocampal region**
**1.1.3.1 Hippocampal formation**
Dentate gyrus	+	4A
Cornu Ammonis (CA) 1-3	++/+++	4A,B	Pyramidal cells, long proc.
Subiculum	+
**1.1.3.2 Para-/retrohippocampal region**
Entorhinal cortex	+
Perirhinal cortex	+
Postrhinal cortex	+
Presubiculum	+
**1.1.4 Basal ganglia**
Striatum
Dorsal region (Caudate putamen)	+++	2A,C,D	Single neurons
Ventral region (Nucleus accumbens)	+++		Single neurons
Islands of Calleja	−
Medial region
(Lateral septal complex)	+/++		Mainly low intensity
Pallidum
Dorsal region
(Globus pallidus med./lat.)	++
Ventral region	+++		Single neurons
Substantia innominate	−
Medial septal complex	(+)
Basal nucleus (Meynert)	+
**1.1.5 Amygdaloid complex and extended amygdala**
Anterior nucleus	−
Posterolateral nucleus	+
Posteromedial nucleus	+
Basolateral nucleus	+
Basomedial nucleus	+
Central amygdaloid nucleus	+
Medial amygdaloid nucleus	+
Bed nucleus of the stria terminalis	++	2A	Few neurons around aca
Piriform cortex	+	2A
**2 Diencephalon (Interbrain)**
**2.1 Hypothalamus**
Periventricular nucleus	+
Suprachiasmatic nucleus	+/++	3A,B
Median preoptic nucleus	+
Supraoptic nucleus	–/+	3A	Intense fibers
Paraventricular nucleus	+	3D	Intense fibers
Dorsomedial nucleus	+	4D
Arcuate nucleus	+
Preoptic area (lat./med.)	+
Anterior hypothalamic area	+
Ventromedial nucleus	+
Posterior hypothalamic area	+
Lateral hypothalamic area	–/+
Tuberal nuclei	–/+
Terete hypothalamic nucleus	+
Median eminence	−	3C	Internal-layer: few axons
Mamillary nucleus	–/+
Pituitary gland (anterior/posterior)	−
**2.2 Thalamus** (Dorsal thalamus)	+		Unstained processes
Anterior group of the dorsal thalamus	+
Medial group of the dorsal thalamus	+
Midline group of the dorsal thalamus	+
Intralaminar nuclei of the dorsal thalamus	+	5A
Lateral group of the dorsal thalamus	+	4D,F
Ventral group of the dorsal thalamus	+	5
**2.3 Metathalamus (Geniculate bodies)**
Medial geniculate body	+
Lateral geniculate body (LGB), dorsal part	–/+		Faint somata
Intergeniculate leaflet of the LGB	–/+		Not explicit visible
Lateral geniculate body, ventral part	+	4E
**2.4 Epithalamus**
Pineal organ	−
Medial habenular nucleus	+++	4A,C,D	Somata, few processes
Lateral habenular nucleus	++	4A,C,D
**2.5 Subthalamus** (Prethalamus, Ventral thalamus)
Subthalamic nucleus	+		Scattered somata
Zona incerta, dorsal part	–/+	5A	Few neuronal somata
Zona incerta, ventral part	+	5A	Many somata
Reticular nucleus of the thalamus	+
**3 Mesencephalon (midbrain)**
**3.1 Tegmentum mesencephali**
Oculomotor nerve nucleus	+		
Accessory oculomotor nerve nucleus	+		Faint somata
(Edinger-Westphal)
Nucleus nervi trochlearis	(+)		Faint somata
Mesencephalic trigeminal nucleus	−		
Substantia nigra, pars compacta	+	5A,D
S.n., pars reticularis	(+)	5A	Scattered somata
Ventral tegmental area	+		
Interpeduncular nucleus, rostral	+	8A	Somata without processes
Interpeduncular nucleus, caudal	(+)	8A	Faint somata, IR puncta
Red nucleus	−		
Periaqueductal gray	+	6A	Somata dorsal > ventral
Ncl. posterior commissure (Darkschewitsch)	+
Interstitial nucleus of Cajal	+		
Laterodorsal tegmental nucleus	+++	7A,B,C	Dense crossing fibers
Laterodorsal tegmental ncl, ventral part	+++	7A,B	Dense fibers, processes
Pedunculopontine tegmental nucleus	+++	6A,D, 7A	Many processes
Microcellular tegmental nucleus	++	6AC	Small neurons no proc.
Deep mesencephalic nucleus	(+)	6A,C	Fibers
**3.2 Pretectum**
Anterior pretectal nucleus	++	5A,B
Olivary pretectal nucleus	+	5A
**3.3 Tectum mesencephali (Lamina tecti)**
Inferior colliculus	−	7A
Superior colliculus	−	6A,B	Dense optic fiber systems
– deep gray layer	(+)	6B	
**4 Rhombencephalon (hindbrain)**
**4.1 Metencephalon**
**4.1.1 Pons**
Cochlear nuclei	+
Vestibular nuclei	+/+++	8B, 9J-K	Many terminals in MVePC
Abducens nucleus	(+)
Facial nucleus	(+)
Reticulotegmental nucleus of the pons	++	6A,F
Pontine nuclei	−	6A
**4.1.2 Cerebellum**
Cortex
Molecular layer	−	8C,E,F	Dense terminals
Purkinje cell layer	−	8C,E,F	Terminals
Granule cell layer	+	8E	Scattered neurons
Deep cerebellar nuclei
Fastigial nucleus	+
Interposed nucleus	+	8C,D	Small somata, no proc.
Dentate nucleus	+
**4.2 Myelencephalon (Medulla oblongata)**
**4.2.1 Sensory regions**			
Principal sensory trigeminal nucleus	(+)
Supratrigeminal nucleus	−		
Cuneate nucleus	(+)		
Superior olivary complex	+	7D	
Dorsal nucleus of the lateral lemniscus	+/++/+++		Fibers reaching laterally
Paralemniscal nucleus	+++		
Parabrachial nucleus	+		
Pre-Bötzinger complex	−		Ventrolateral medulla
**4.2.2 Motor regions**
Nucleus ambiguous	(+)
Hypoglossal nucleus	(+)
Superior/inf. salivarynucleus (-salivatory ncl.)
Motor nucleus of the trigeminal nerve	−
MTT motoneurons	−		
Inferior olivary complex	(+)
Paramedian reticular nucleus	(+)
Locus ceruleus	+
Raphe nuclei, dorsal	++	6A,E
Raphe nuclei, dorsal (caudal part)	(+)	7C
ventral	+	6A	
median/paramedian	+	6A
Kölliker-Fuse-nucleus (subparabrachial ncl.)	+
**5. Spinal cord**
Anterior horn	+		Large motoneurons
Posterior horn	+		Small neurons, no afferents
Lateral horn	+		Some pregangl. neurons
Central gray	+		Scattered neurons
**6. Ganglia**
Trigeminal ganglion (sensory)	−		
Spinal ganglion (sensory)	−		
Superior cervivcal ganglion (sympathetic)	−		Few fibers
**7. Fiber tracts** (alphabetical order)
Anterior commissure	−	2A	
Central tegmental tract	−		
Corpus callosum	−	2A	
Fasciculus retroflexus (Meynert)	+++	4C,D, 5A,C
Lateral lemniscus	−		
Longitudinal fasciculus of pons	−	6A	
Mamillothalamic tract	−	4F	
Medial lemniscus	−	4E, 6A	
Medial longitudinal fasciculus	−	6A	No fibers
Olfactory nerve	+	1A	
Optic tract	++	4E
Posterior commissure	−	5A	Dorsal to 3V
Stria medullaris thalami	−		
Subthalamic fasciculus	−		

*^1^Relative levels of neuronal somata immunoreactivity: − not detectable, (+) faintly labeled, + low, ++ moderate, +++ intense*.

The Adobe Photoshop program was used to convert color- to black-and-white images, to adjust image contrast and brightness and to add labels. These do thus not necessarily provide intensity information since the b/w-images were increased in contrast and/or brightness to better visualize IR structures (i.e., neuronal somata and processes) for publication.

Brain regions were identified according to the stereotaxic atlas of the mouse brain (Paxinos and Franklin, 2013) and the overviews given in chapters of “The Mouse Nervous System” by Watson (2012).

Control incubations, carried out by omitting primary or secondary antibodies, showed the absence of the fluorescent signal. Cygb antibody specificity was tested by preabsorption of the primary antibody with the synthetic immunogen (5, 10, or 15 μg recombinant protein per ml) overnight at RT and gentle agitation. Brain sections were then incubated in the solution, as described above. Immunoreaction was abolished when the antigen concentration was 0.5 nmol or higher. In addition, dye-swap experiments demonstrated that antibody-staining patterns (cytoplasmic/nuclear) were independent of the fluorescent dye.

## Results

**Figure 1:**
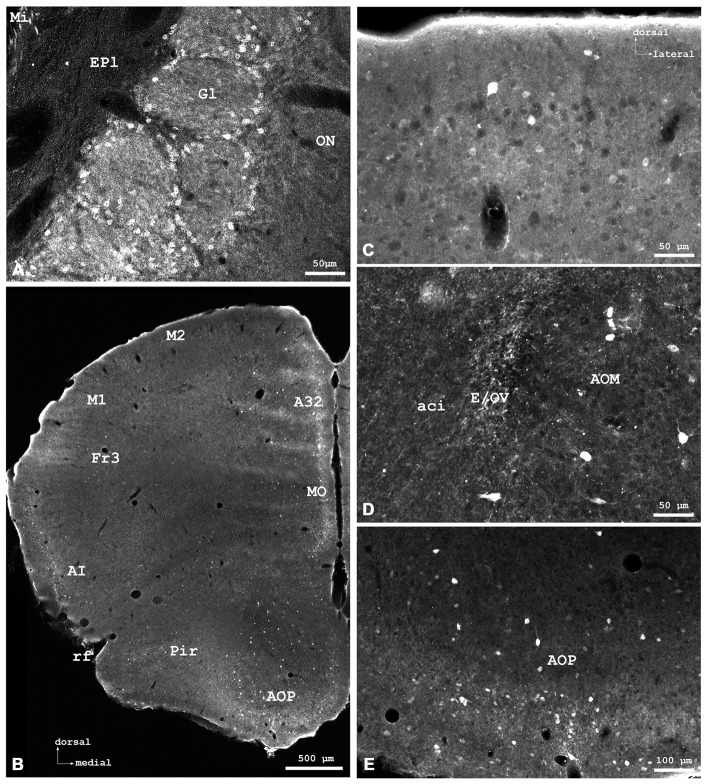
**Cytoglobin (Cygb)-immunofluorescence in frontal sections of the mouse brain. (A)** Cygb-immunoreactive (Cygb-IR) periglomerular neurons in the glomerular layer (Gl) of the olfactory bulb, at the level of approximately interaural +8 mm, corresponding to Panel of the mouse brain atlas. **(B)** Low power photomicrograph “multiple image alignment” (MIA) showing orbital regions of the cerebral cortex (CCo) at the level of approximately interaural +6.0 mm, corresponding to Figure 12 of the mouse brain atlas. **(C)** Higher magnification of the section shown in **(B)** demonstrating strong and faintly labeled neurons in the secondary motor cortex. **(D)** Higher magnification from **(B)** showing sparse neurons and fibers in the anterior olfactory region. **(E)** Higher magnification from **(B)** demonstrating Cygb-neurons in the posterior part of the anterior olfactory area (AOP). Abbreviations: A32, cingulate cortex; ACI, intrabulbar part of the anterior commissure; AI, agranular insular cortex; AOM, medial part of the AOP; E/OV, ependyma of the olfactory ventricle; EPl, external plexiform layer of the olfactory bulb; M1/M2, primary/secondary motor cortex; Fr3, frontal cortex; Mi, mitral cell layer; MO, medial orbital cortex; ON, olfactory nerve layer; Pir, piriform cortex; rf, rhinal fissure.

**Figure 2:**
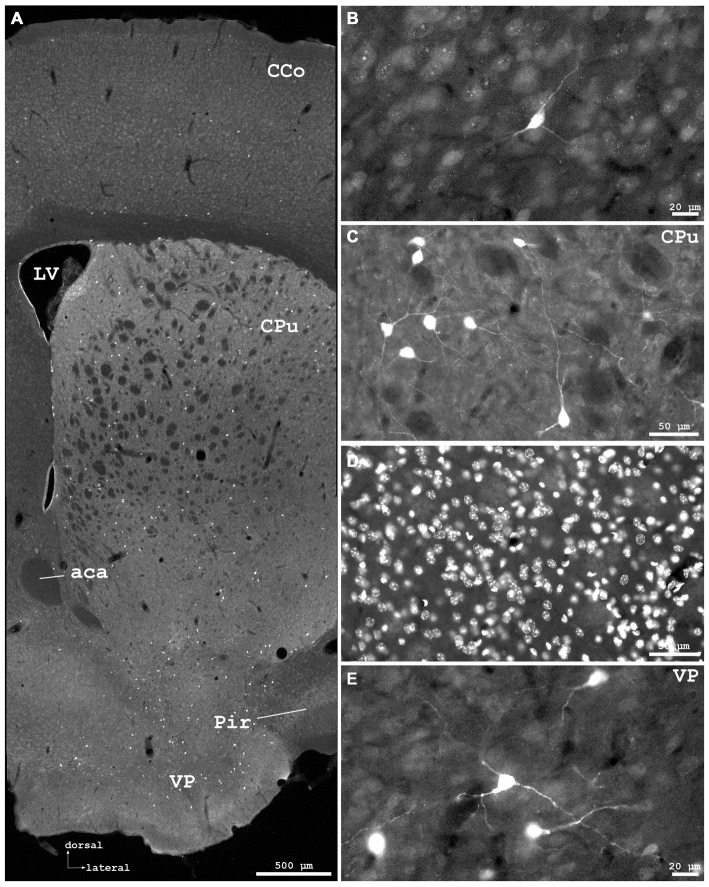
**Cygb-immunofluorescence in a frontal section of the mouse brain at the level of approximately interaural +4.3 mm, corresponding to Figure 27 of the mouse brain atlas. (A)** Low power photomicrograph (MIA) showing primary motor and somatosensory regions of the CCo, lateral ventricle (LV), striatum (Ncl. caudatus/putamen, CPu) and ventral pallidum (VP). **(B)** Higher magnification demonstrating Cygb-IR neuron in lamina IV of S1 cortex, **(C)** Multipolar neurons in the lateral striatum, **(D)** DAPI nuclear staining of the same sector, and **(E)** multipolar neurons with long processes in the VP. Abbreviation: ACA, anterior part of the anterior commissure; Pir, piriform cortex.

**Figure 3:**
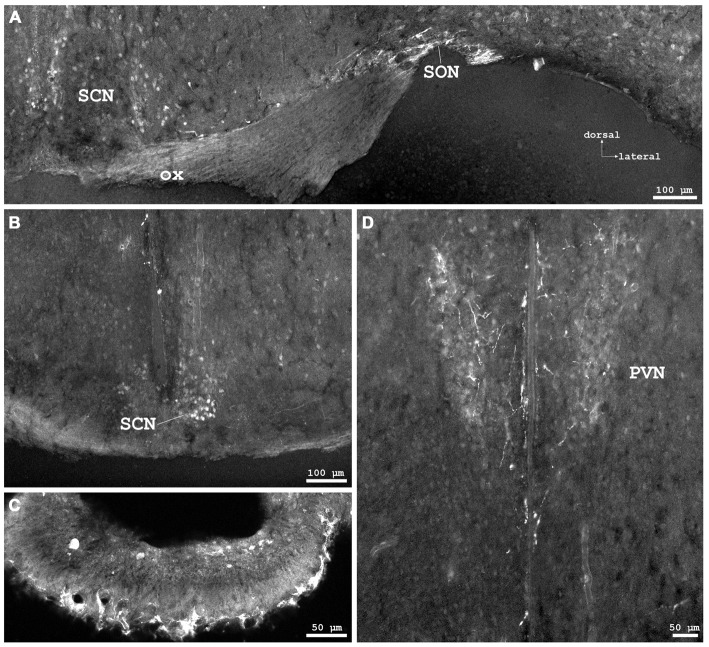
**Cygb-immunofluorescence in a frontal section of the mouse brain at the levels of approximately interaural +3.3–3.1 mm, corresponding to Figures 35–37 of the mouse brain atlas. (A)** Overview (MIA) showing the optic chiasm (ox) and basal hypothalamus. Labeled cells were found in the supraoptic nucleus (SON) and in core and shell regions of the medial suprachiasmatic nucleus (SCN). In the posterior SCN **(B)**, neurons labeled with various strength were observed within the borders of the nucleus. **(C)** Median eminence: few small IR structures, mainly processes, were seen.** (D)** Faintly labeled neurons in the anterior magnocellular part of the hypothalamic paraventricular nucleus (PVN), where stronger labeled processes (probably axons) were present.

**Figure 4:**
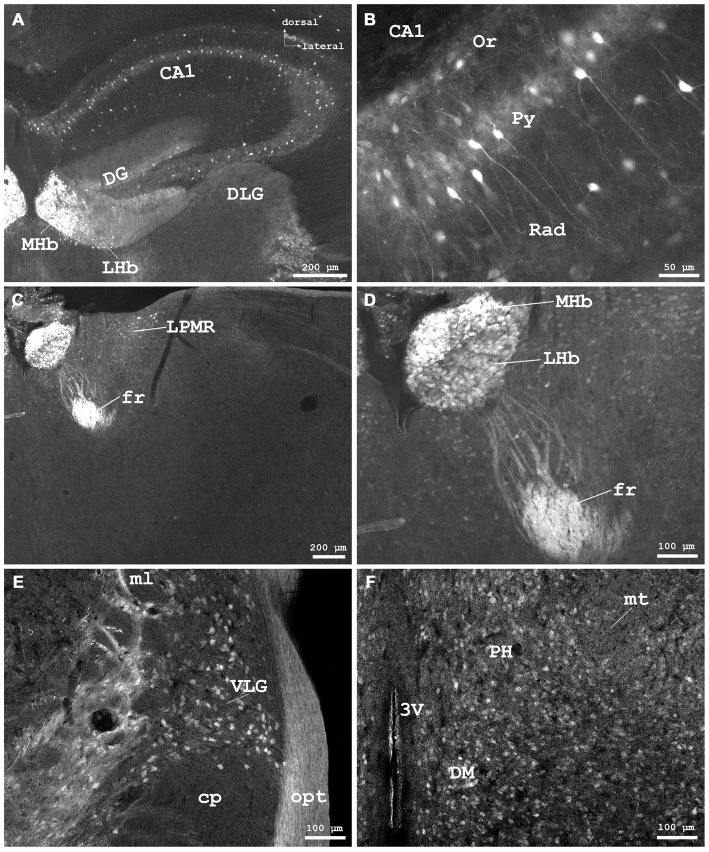
**Cygb-immunofluorescence in a frontal section of the mouse brain. (A)** Habenular nuclei and anterior hippocampal formation at the level of approximately interaural +2.1 mm, corresponding to Figure 45 of the mouse brain atlas. Strong staining of neurons in the medial habenular nuclei (MHb), while those in the lateral nuclei (LHb) were less stained. **(B)** Higher magnification from the medial CA1 region of the hippocampus: strong immunostaining of neurons in the pyramidal cell layer (Py). A few neurons from the oriens layer (Or) and stratum radiatum (Rad) were also stained. **(C)** Habenular nuclei and fasciculus retroflexus (fr) at the level of approximately interaural +1.7 mm, corresponding to Figure 48 of the mouse brain atlas, **(D)** Higher magnification demonstrating fibers entering the fasciculus retroflexus. **(E)** ventral aspect of the lateral geniculate nucleus (VLG) between the optic tract (opt) and the medial lemniscus (ml). **(F)** Scattered neurons in the posterior hypothalamic area (PH) and the dorsomedial hypothalamic nucleus (DM). Abbreviations: 3V, third ventricle; CA1, Cornu ammonis 1; cp, cerebral peduncle; DG, dentate gyrus; DLG, dorsal lateral geniculate nucleus; LPMR, lateral posterior thalamic nucleus, mediorostral part; mt, mamillothalamic tract.

**Figure 5:**
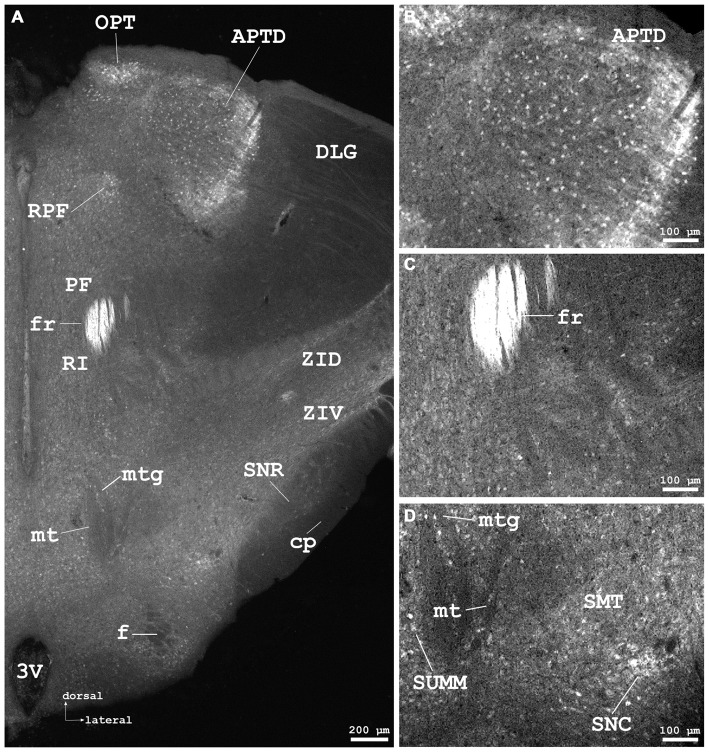
**Cygb-immunofluorescence in a frontal section of the mouse brain at the level of approximately interaural +1.3 mm corresponding to Figure 52 of the mouse brain atlas. (A)** Low power photomicrograph (MIA) demonstrating labeled neurons in the dorsal part of the anterior pretectal nucleus (APTD), olivary pretectal nucleus (OPT), retroparafascicular nucleus (RPF), dorsal and ventral parts of the zona incerta (ZID, ZIV), and substantia nigra pars compacta (SNC) and pars reticularis (SNR). Note that the mamillothalamic tract (mt), fornix (f), mamillotegmental tract (mtg) and cerebral peduncle (cp) are unstained, while the fasciculus retroflexus exhibits strong Cygb-immunoreactivity. Moderately labeled neurons were present in neighboring regions such as the parafascicular thalamic nucleus (PF) and the rostral interstitial (RI) nucleus of the medial longitudinal fasciculus (MLF). **(B)** Higher magnification of IR neurons in the APTD. **(C)** IR fibers in high density are seen in the fasciculus retroflexus (fr). **(D)** Higher magnification showing the mamillothalamic (mt) and mamillotegmental (mtg) tracts, and stained neurons of the SNC. Abbreviations: 3V, third ventricle; SMT, submamillothalamic nucleus; SuMM, submamillary nucleus, medial part.

**Figure 6:**
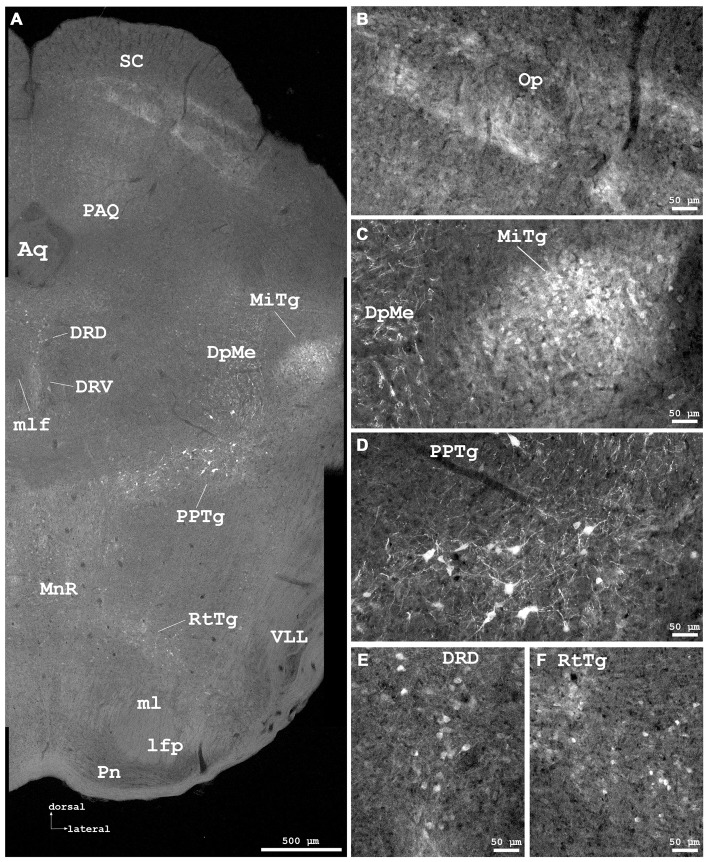
**Cygb-immunofluorescence in a frontal section of the mouse brain at the level of approximately interaural −0.4 mm, corresponding to Figure 66 of the mouse brain atlas. (A)** Low power photomicrograph (MIA) showing superior colliculus (SC), dorsal raphe and tegmental nuclei. **(B–F)** Higher magnifications from **(A)** demonstrating Cygb-IR neuronal structures. **(B)** Faintly labeled fiber systems and neuronal somata in the optic nerve layer of the SC (Op). **(C)** Small neurons with only few processes are found in the microcellular tegmental nucleus (MiTg), fiber systems in the deep mesencephalic nucleus (DpMe). **(D)** Large neurons with multiple processes are present in the pedunculopontine tegmental nucleus (PPTg). **(E)** IR neurons in the dorsal and ventral aspects of the dorsal raphe (DRD, DRV), and **(F)** in the reticulotegmental nucleus of the pons (RtTg). Abbreviations: Aq, aquaeductus cerebri; lfp, longitudinal fascicle of the pons; ml, medial lemniscus; mlf, medial longitudinal lemniscus; MnR, median raphe nucleus; PAQ, periaqueductal gray; Pn, pontine nuclei; VLL, ventral nucleus of the lateral lemniscus.

**Figure 7:**
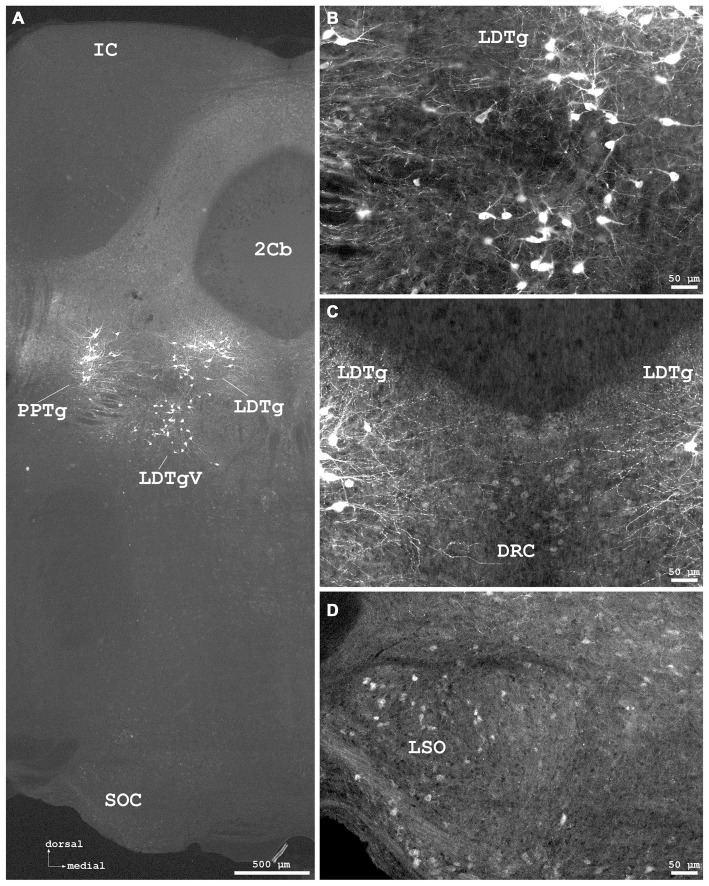
**Cygb-immunofluorescence in a frontal section of the mouse brain at the levels of approximately interaural −1.0 to −1.3 mm, corresponding to Figures 71–73 of the mouse brain atlas. (A)** Low power photomicrograph (MIA) showing the inferior colliculus (IC), cerebellar lobule 2 (2Cb), tegmental nuclei and the superior olivary complex (SOC). **(B)** Higher magnifications “extended focal imaging” (EFI) demonstrating Cygb-IR neurons in the laterodorsal tegmental nucleus (LDTg), in its ventral part (LDTgV) and in the PPTg. **(C)** LDTg nucleus neurons and fibers crossing contralaterally EFI. Note the light staining of neurons in the caudal part of dorsal raphe neurons (DRC). **(D)** Some neurons in the lateral superior olivary nucleus (LSO) were stained.

**Figure 8:**
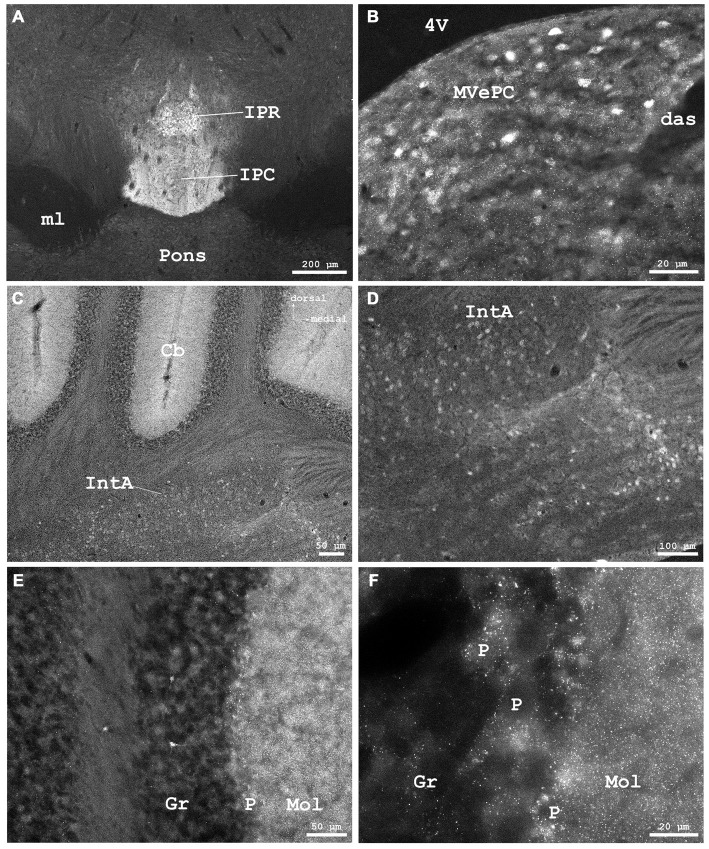
**(A)** Cygb-immunofluorescence in a frontal section of the mouse brain at the level of approximately interaural 0, corresponding to Figure 62 of the mouse brain atlas showing labeled neurons in the rostral part of the interpeduncular nucleus (IPR) and labeled terminals in its caudal part (IPC), **(B–F)** were taken from a frontal section of the mouse brain at the level of approximately interaural −2.4, corresponding to Figure 83 of the mouse brain atlas. **(B)** IR neurons and terminals in the medial vestibular nucleus, parvocellular part (MVePC). **(C)** Low power photomicrograph demonstrating cortical (upper part) and medullary regions (lower part) of the cerebellum (Cb). **(D)** Higher magnification of IR neurons in the anterior cerebellar nucleus interpositus (IntA). **(E)** Higher magnification from cerebellar cortex demonstrating IR neurons in the granular layer (Gr). IR puncta are present throughout the molecular (Mol) and Purkinje cell (P) layers. **(F)** Higher magnification demonstrating dense IR puncta surrounding unlabeled Purkinje cell somata (P) of the ganglionar cell layer. Abbreviations: 4V, fourth ventricle; das, dorsal acoustic stria; ml, medial lemniscus.

**Figure 9:**
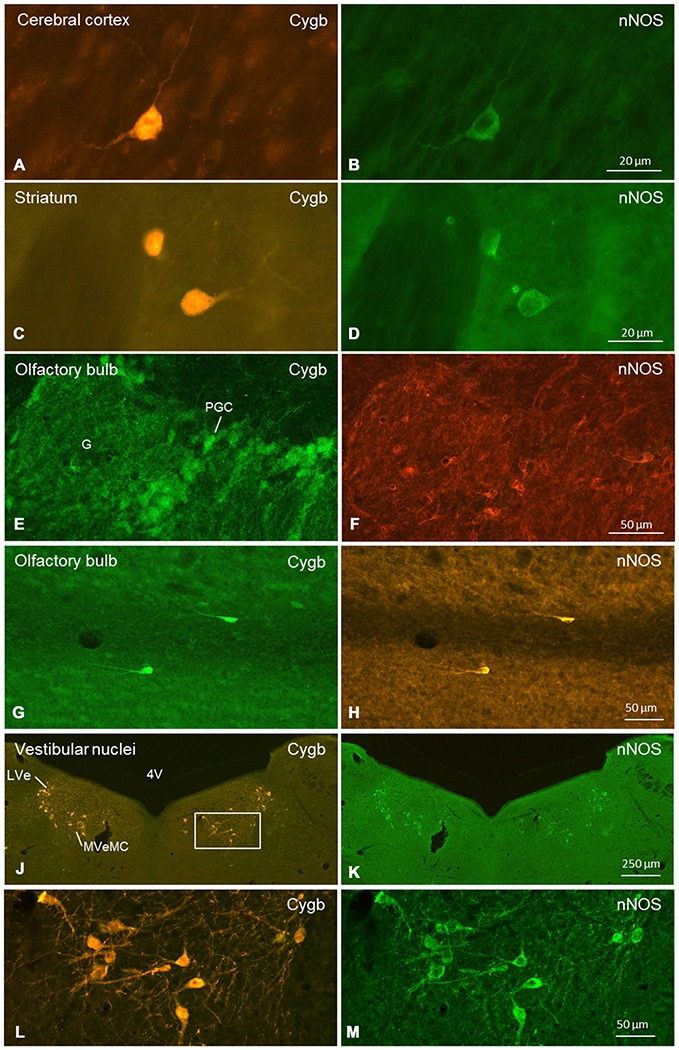
**Double-immunofluorescent labeling of cytoglobin (Cygb, left row) and (nNOS, right row) in selected regions of the mouse brain (frontal sections).** Complete or partial colocalization of both substances was observed in the CCo **(A,B)**, striatum **(C,D)**, glomerular layer **(E,F)** and subependymal layer **(G,H)** of the olfactory bulb, and medial and lateral vestibular nucleus **(J–M)**. The boxed area in **(J)** is shown in higher magnification in **(L)**. Note that **(E–H)** stem from a dye-swap experiment. Abbreviations: 4V, fourth verntricle; G, glomerulus; PGC, periglomerular cells; LVe, lateral vestibular nucleus; MVeMC, medial vestibular nucleus, magnocellular part.

Cygb-immunoreactivity was found in many regions throughout the mouse brain. The signals were, in all cases, seen in the whole neuronal soma, i.e., in the nucleus and the cytoplasm (Figures 1–8). This was clearly confirmed by double-staining in which the Cygb-signal was compared to the distribution of, e.g., neuronal NO-synthase (the latter restricted to the perikaryon; Figure 9).

The immunofluorescent signals were of variable intensity ranging from weak (and just above background) to very intense. In a given cell group, the appearance of staining was basically identical between animals, i.e., there were no significant differences in distribution pattern, amount or intensity detected by immunofluorescence. In addition, the staining patterns appeared similar between female and male animals. Figures 1–8, arranged in predominantly anterior-posterior order, present typical frontal brain sections from an adult male mouse. All approximate interaural levels and corresponding figure numbers given in the legends relate to the mouse brain atlas by Paxinos and Franklin (2013). The orientation of sections is given in the low power panels in the figures, and all higher magnifications are oriented in the same manner. Table 1 provides the relative intensity of Cygb-immunoreactivity levels of neuronal somata in the studied regions, and gives information on noticeable appearance of neuronal dendritic and axonal processes. In the following, we will present important aspects of Cygb-immunoreactivity in major brain regions in a roughly rostro-caudal sequence.

### Telencephalon

Neurons exhibiting mainly intense staining intensity were found in several parts of the **cerebrum**, i.e., the cerebral cortex (CCo), olfactory bulb, hippocampal formation, basal ganglia and amygdala.

### Olfactory Bulb

The (peri)glomerular regions exhibited high levels of immunoreactivity (Figure 1A). Glomeruli exhibited a dense association of punctate IR structures, and many small periglomerular neurons were labeled by the antibody. Single neurons were stained in the granule and plexiform layers. The subependymal cell layer exhibited some strongly stained large neurons that most probably are deep short-axon cells. In the medial and posterior parts of the anterior olfactory area (AOP), neuronal cell bodies with few processes were observed (Figures 1B,D,E).

### Cerebral Cortex

Stained neurons were found in all laminae, as exemplarily shown in Figure 2A. They amounted to roughly less than 10% of the total of neurons. Immunoreactive (IR) neurons were observed throughout all cortical areae. Figure 2B depicts that these neurons are relatively large (up to 20 μm in soma diameter) and equipped with distinct processes. Immunofluorescent dots were seen covering unlabeled somata (Figure 2B). In the agranular insular cortex (AI in Figure 1B), scattered neurons were concentrated in deeper cortical layers, while in the frontal cortex (Fr3 in Figure 1B) strongly stained terminals stood out rather than stained neuronal cell bodies. In medial parts of cingulate cortex (A32 in Figure 1B), in the piriform (Pir in Figure 2A), frontal and orbital cortices, in primary and secondary motor area as shown in Figures 1B, 2A, no layer predominance was obvious.

In the **hippocampal formation**, strongly stained pyramidal cells with apical and long basal processes were seen in CA1–3 (Py in Figure 4B). Cell bodies in the dentate gyrus (DG in Figure 4A) and in the subiculum were less IR. This was also the case for somata in the para- and retrohippocampal region, i.e., the presubiculum, ento-, peri-, and postrhinal cortex.

### Basal Ganglia

In the rostral aspects, the basal nucleus (Meynert), as well as in the dorsal **striatum** (caudate putamen, CPu in Figure 2A), scattered multipolar neurons were observed throughout the anterior-posterior extent. They were seen more often in the medial regions than laterally, and their processes were larger (up to 300 μm in the section level, see Figure 2C). These processes were prominent in the striatum, in particular in its dorsal and lateral parts (Figures 2A,C). The increased neuropil staining probably accounts for the higher background in this region compared to the vicinity, e.g., the CCo (Figure 2A). The typical fiber systems, cross-cut in the frontal sections, were not immunofluorescent (see higher magnification in Figure 2C). The comparison of Cygb-immunolabeling (Figure 2C) with nuclear DAPI-staining (Figure 2D) showed that total cells outnumbered Cygb-neurons by far. Multipolar neuronal somata of approximately 15 μm diameter with labeled processes made up to less than ten percent of neurons in this region. In the shell and core regions of the accumbens nucleus (encircling the anterior commissure in sections approximately interaural +5 mm; not shown), single strongly stained somata with processes were seen. Islands of Calleja were not apparent. Neuronal cell bodies with processes of 700 μm length were labeled by the antibody in the dorsal and in the ventral pallidum (VP; Figures 2A,E). In the medial septal nucleus of the pallidum and in the vertical limb of the nucleus of diagonal band of Broca, faintly labeled neurons were seen. In the septohypothalamic nucleus (also termed part of the lateral septal nucleus), located ventromedial to the (unstained) anterior commissure (aca in Figure 2A), moderately stained neuronal somata without visible processes were present.

### Amygdaloid Complex and Extended Amygdala

The central, medial and lateral nuclei (LHb) exhibited IR neuronal somata that did, in most cases, not exhibit processes. Some stronger stained cells were seen in the various aspects of the bed nucleus of the stria terminalis (BNST; surrounding the anterior commissure). Neurons in the piriform cortex were only faintly stained (although immunofluorescence was clearly above background).

### Diencephalon

Distinct IR structures were seen in all parts. In the **hypothalamus**, the core-region of the medially-located suprachiasmatic nucleus (SCN) was nearly free of immunostained neurons, while in the shell-region small somata were labeled (SCN in Figure 3A). In the posterior SCN as well as in the subparaventricular zone, immunofluorescent neurons were present (Figure 3B). In the paraventricular nucleus (PVN), faintly labeled neurons were observed in the parvocellular (medial) but not in the magnocellular part (PVN in Figure 3D). The supraoptic nucleus (SON) was nearly devoid of labeled neurons (SON in Figure 3A). Other regions such as dorso- and ventromedial hypothalamic nuclei exhibited moderately labeled somata (see Table 1). Few small neurons were stained in the internal layer of the median eminence, while some IR fibers were seen in its external layer (Figure 3B). IR fibers were seen in the optic chiasm, ventral SCN and explicit, in SON and PVN (Figure 3). Neither the anterior nor the posterior part of the pituitary gland exhibited IR structures.

### Thalamus (Dorsal Thalamus)

Neuronal somata in various groups (anterior, medial, midline, intralaminar, lateral and ventral) exhibited faint to moderate immunoreactivity without apparent differences between regions. Fluorescence was restricted to neuronal somata and not found in processes in these regions. Most neurons in the lateroposterior thalamic nucleus (LPMR in Figure 4C) were faintly stained, while in its lateral aspects some cells with stronger fluorescence were seen. The mediodorsal thalamic nucleus exhibited moderately immunofluorescent neuronal somata in its lateral, but not in its medial part. In posterior thalamic regions, the retroparafascicular nucleus (RPF in Figure 5A) and the parafascicular thalamic nucleus (PF in Figure 5A), a part of the intralaminar thalamic group, as well as the posterior thalamic nucleus exhibited very faint to moderate neuronal Cygb-immunofluorescence.

Neurons were not seen in the ventral posteromedial thalamic nucleus (VPM) and the ventral posterolateral thalamic nucleus (VPL) where IR fibers were present. Some neurons with faint to moderate fluorescence were observed in the ventrolateral and in the ventromedial thalamic nuclei. In the latter, fibers extending to the VPL were observed. Neuronal somata in the posterior thalamic nuclear group (lateral and ventral to anterior pretectal nucleus (APTD) in Figure 5A) were mainly unstained.

### Metathalamus

The medial geniculate body and the ventral part of the lateral geniculate body (LGB; VLG in Figure 4E) exhibited moderately stained somata without processes. The dorsal lateral geniculate exhibited only few neurons that barely exceeded background. Cygb-immunoreactivity was not noticeable in the intergeniculate leaflet.

### Epithalamus

The pineal gland did not exhibit IR structures. The medial habenular nuclei (MHb) exhibited strong immunofluorescent staining of dense-packed neuronal cell bodies, while labeling was less intensive in the lateral habenular nuclei (Figures 4A,C,D). IR fibers were not detected in the stria medullaris, in the epithalamic (=posterior) and in the habenular commissures.

#### Subthalamus (Ventral Thalamus)

Scattered somata of moderate immunoreactivity were observed in the subthalamic nucleus. In the ventral part of the zona incerta (ZIV in Figure 5A), many moderately stained neurons were seen, to smaller numbers in its dorsal part (ZID). Moderately stained neurons were seen in the reticular thalamic nucleus.

#### Mesencephalon (Midbrain)

IR neuronal somata were found in tegmental, pretectal and tectal structures. In the **tegmentum**, striking groups of strongly stained neurons with long processes were found in the laterodorsal tegmental nucleus (LDTg) including its ventral part (Figures 7A–C) where also dense fiber systems were observed. Some immunofluorescent fibers crossed to the contralateral nucleus (Figure 7C). Similar remarkable neurons were found in the pedunculopontine tegmental nucleus (PPTg; Figures 6A,D, 7A). The substantia nigra exhibited moderately stained neurons without processes, predominantly in the pars compacta, and less in the pars reticularis (Figures 5A,D).

The ventral tegmental area and the nucleus of the posterior commissure (Darkschewitsch) exhibited moderately stained neuronal somata, while (magnocellular) neurons of the red nucleus were unstained. The periaqueductal gray exhibited moderately fluorescent neurons. Fibers of the central tegmental tract were unstained, as were those of the medial longitudinal fascicle (MLF in Figure 6A). The rostral interstitial nucleus of the MLF (RI in Figures 5A,C) exhibited moderately IR neurons. The interpeduncular nucleus (IPR), rostral part (IPR in Figure 8A) showed moderately labeled neuronal somata, while these were only faintly seen in its caudal part (IPC) where IR puncta in high density were present. The intermediate and lateral interpeduncular subnuclei were unstained (Figure 8A). Faint neurons but clearly labeled IR fiber systems were observed in the deep mesencephalic nucleus (DpMe in Figures 6A,C).

#### Pretectum

The anterior pretectal nucleus (APTD in Figures 5A,B) and the olivary pretectal nucleus (OPT in Figures 5A,B) exhibited clearly immunostained neuronal somata as well as labeled neuropil.

#### Tectum

In the superior colliculus (SC; Figure 6A), faintly labeled neurons were observed in the deep gray layer, while no IR structures were seen in the zonal and superficial gray layers. Fibers were immunolabeled in the optic layer (Op in Figure 6B). The inferior colliculus (IC) was largely devoid of IR structures (Figure 7A).

#### Rhombencephalon (Hindbrain)

Immunofluorescence was detected in metencephalic structures (pons, cerebellum) and in the myelencephalon. In the **pons**, neurons of the pontine nuclei (PN in Figure 6A) remained unlabeled by the antibody, as was the longitudinal fascicle of the pons (LFP in Figure 6A). The cerebral peduncle (basal part, CP in Figures 4E, 5A) was unstained.

Neurons of the abducens nerve nucleus and those in the facial nerve nucleus were rather faintly stained; the facial nerve was widely devoid of IR fibers. Neuronal somata of the cochlear nuclei showed moderate staining. The medial vestibular nuclei exhibited different immunolabeling. I.e., the parvocellular region (MVePC) exhibited moderately stained neuronal somata and many immunofluorescent puncta (Figure 8B), while neurons of the magnocellular region of the medial vestibular nucleus (MVePM in Figures 9G,I) and the lateral vestibular nucleus (LVe in Figure 9) exhibited strong immunostaining. The nucleus prepositus (medial to the MVeMC) was unstained.

#### Cerebellum

As seen in Figures 8A,C,E, the molecular layer (Mol in Figure 8E) exhibited IR puncta in high density, while IR neuronal somata were not observed. Cortical Purkinje-neurons (P in Figure 8F) were Cygb-negative while IR puncta were seen in their close vicinity. Single neurons were stained in the granular layer (Gr in Figure 8E). The deep cerebellar nuclei showed some weakly labeled neurons and IR terminals in their vicinity. The anterior interposed nucleus (IntA) of the cerebellum is demonstrated in Figures 8C,D.

#### Myelencephalon: Medulla Oblongata

Neurons of the principal sensory nucleus of the trigeminal nerve, those of its motor nucleus as well as the pseudounipolar neurons of the mesencephalic nucleus of the trigeminal nerve (part of the trigeminal ganglion) were unstained. In the dorsal aspects of the raphe nuclei (DRD in Figures 6A,E), neurons exhibited clear immunolabeling. In its ventral aspects, moderate staining was found, that was even less intensive in the median and paramedian raphe neurons.

While in the dorsal nucleus of the lateral lemniscus (DLL) moderate to strong immunostaining of neurons was observed, cells in the ventral nucleus of the lateral lemniscus (VLL in Figure 6A) were unstained.

In the **spinal cord** (not shown), moderately immunofluorescent neurons without distinct processes were seen in the gray matter. Large IR motoneurons were observed in the anterior horn, small somata in the posterior horn and medium-sized neurons in the lateral horn as well as in the central gray.

#### Peripheral Ganglia

Cygb was not observed in neurons of the peripheral ganglia such as the sensory trigeminal and spinal ganglion or the sympathetic superior cervical ganglion. In the latter, few IR fibers were seen.

#### Double-Staining Experiments

We conducted a more punctual study on the general question whether Cygb-positive neurons colocalized neuroactive substances. Tested were tyrosine-hydroxylase (TH) and nNOS. There was no double-labeling for Cygb and TH in the present study, although both proteins were detected in the same brain sections. In contrast, many neurons exhibiting Cygb-immunoreactivity were also nNOS-positive. In some regions (e.g., CCo, striatum, vestibular brainstem nuclei; see Figure 9), we observed a one-hundred-percent overlap of both immunoreactivities, i.e., every Cygb-neuron was nNOS-IR and* vice versa*. In other parts of the mouse brain, e.g., periglomerular cells of the olfactory bulb, it appeared that not all Cygb-neurons were nNOS-positive. However, it is possible that nNOS-expression in these cells was below the immunofluorescence detection level. Finally, some neuronal groups (cerebellar cortex Purkinje-cells, for example) exhibited neither nNOS- nor Cygb-immunofluorescence.

## Discussion

Cygb was initially identified as STAP in the mammalian liver (Kawada et al., 2001), but later studies showed a much broader distribution in many different tissues (Asahina et al., 2002; Burmester et al., 2002; Trent and Hargrove, 2002). This is due to the expression of Cygb in fibroblasts and related cell types (Nakatani et al., 2004; Schmidt et al., 2004). Most previous analyses focused on the cellular localization and the function of Cygb in cells of the supportive tissues, leading to the conclusion that modulated Cygb-expression is associated with diseases such as fibrosis or cancer (He et al., 2011; Oleksiewicz et al., 2013; Kawada, 2015; Thuy Le et al., 2015). There is, however, conclusive evidence that Cygb also resides in neurons (Schmidt et al., 2004, 2005; Hundahl et al., 2010, 2014). In contrast to the localization in fibroblast-related cells, Cygb was present in both, cytoplasm and nucleus of neurons. Thus the present study was conducted to provide a comprehensive analysis of Cygb-IR neurons in the nervous system of the mouse. Although some peripheral ganglia were tested, we concentrated on neurons of the central nervous system. Frontal sections of the perfusion-fixed mouse brain were exposed to an antibody directed against Cygb (Schmidt et al., 2004), and the immunoreaction was visualized with fluorescence-coupled secondary antibodies.

We focused on the following questions: (1) Which neurons/neuronal groups are Cygb-IR; (2) which neurons exhibit immunolabeled processes; and (3) which of the various fiber tracts were labeled by the antibody. We found that in all labeled neurons, both the perikaryon (cytoplasm) and the nucleus were Cygb-IR, while fibroblast-related cells showed only cytoplasmic immunofluorescence. This agrees with our previous studies (Schmidt et al., 2004; Avivi et al., 2010). The same intracellular localization was also obvious in images of hippocampal neurons (Hundahl et al., 2010), although the authors mentioned Cygb-immunoreactivity only to be present “in the perikarya, dendrites and axons of the neuron”. This particular location of Cygb was presently also evident in our double-immunofluorescent stainings showing Cygb in cytoplasm and nucleus, but nNOS only in the cytoplasm of the same neuronal cells (see Figure 9).

### Widespread and Differential Cygb-Expression in Mouse Brain

Cygb-IR neurons were observed in all brain regions to different amounts and with different strengths of immunofluorescent staining, however, with constant, reproducible expression between animals, and regardless of their sex. While the present analysis was in preparation, Hundahl et al. (2010) reported the widespread distribution of Cygb-mRNA and -protein in mouse brain. Notably, the datasets from both studies coincide well regardless of different antibodies and detection methods. For example, typical aspects of the distribution pattern such as strong staining of tegmental neurons, the intense labeling of habenular nuclei and Meynert’s tract as well as the absence of staining in cerebellar Purkinje-cells are evident in both studies. Our present study, however, features a more systematic and detailed analysis and presentation of Cygb-positive neurons in the mouse brain under a functional aspect. Table 1 summarizes the localization and intensity of labeling for Cygb-neuronal somata and processes of the mouse brain (including spinal cord, ganglia and fiber systems) in detail and refers to the respective figures, which demonstrate typical examples.

Concerning the first question, it was obvious that Cygb-neurons made up only a small part of the neuronal population in many brain regions. This was observed, for example, in the CCo and the striatum. Neurons in these regions were intensely labeled and exhibited distinctly labeled processes. Striking are those in the tegmental nuclei (e.g., the pedunculopontine and laterodorsal tegmentum), where they exhibited long processes (presumably axons) that project to various brain sites including thalamus, pontine and medullary reticular formation (cf. Saper and Stornetta, 2015). These processes were also seen to cross the cerebral midline. Noticeable are, furthermore, hippocampal pyramidal neurons with dendritic bundles in the strata oriens and radiatum and long basal processes that may represent branching axons projecting to septum, amygdala, and entorhinal cortex areas (Arszovszki et al., 2014). These distinctly Cygb-labeled neurons were also recently described by Hundahl et al. (2014).

Neurons of the thalamus were representative of a second category of Cygb-IR neuronal somata. They exhibited fluorescence of different strength and were not equipped with labeled processes. Despite the rich structural subdivisions of the thalamus and their functional diversity (about 50 distinct nuclei competent for sensory control up to cortical and subcortical feedback loops, cf. Puelles et al., 2012), IR thalamic neurons were of rather similar (faint to moderate fluorescent) appearance. Similarly labeled somata were seen in other brain regions with different functions, e.g., the subthalamus (part of the basal ganglia motor system), periaqueductal gray (primary control center for descending pain modulation), cerebellar nuclei (motor output relay), and anterior and posterior hypothalamic nuclei (sleep- and thermoregulation). More intensely stained somata without labeled processes were found, for example, scattered in the superior olivary complex (SOC) or densely packed in the microcellular tegmental nucleus and in the medial habenular nucleus (present study; Hundahl et al., 2010). Axons of the latter group in these regions provide the habenulo-interpeduncular tract that efferently connects the epithalamic habenular nuclei to the mesencephalic interpeduncular nuclei. This tract, also known as fasciculus retroflexus (Meynert), was strongly labeled in both, the present and the earlier study (Hundahl et al., 2010), while the stria medullaris, providing septal and hypothalamic afferents to the habenular nuclei, was not stained.

### Labeled and Unlabeled Fiber Tracts

Actually, from the various fiber tracts investigated here, only the Meynert-fascicle and the olfactory and optic nerves and tracts were labeled by the Cygb-antibody. All other fiber systems including interhemispheric connections (e.g., corpus callosum), projection systems (e.g., medial and lateral lemniscus and stria medullaris thalami) and association system fibers (connecting neighboring cortical areae) were not labeled. As shown in the figures, devoid of Cygb-immunoreactivity were the mamillothalamic tract, the central tegmental tract with its ascending (sensory) and descending (motor) fibers, the dorsal and MLF of the pons (connecting eye muscle motor nuclei), the anterior commissure (connecting both temporal lobes), the CP consisting of massive fiber systems connecting the CCo with brainstem and pons, and the epithalamic (=posterior) commissure, which is part of the bilateral pupillary reflex chain. The latter is made up from neuronal fibers originating from the (Cygb-negative) neurons of the nucleus of the posterior commissure (also known as nucleus of Darkschewitsch). While it is not surprising that Cygb-negative somata give rise to unlabeled fiber tracts, we also observed unlabeled fibers originating from Cygb-positive neurons, e.g., the habenular commissure connecting the (Cygb-positive) bilateral habenular nuclei to each other, and the medial posterior fasciculus containing axons from labeled neurons in the interstitial nucleus of Cajal.

Taken together, labeled fibers seem to originate exclusively from labeled neurons, while labeled neurons may give rise to unlabeled processes as well.

### Sensory Systems

Neural structures involved in the processing of olfaction showed diverse Cygb-immunolabeling. Within the olfactory bulb, glomeruli exhibited a dense network of labeled axons and dendrites originating from olfactory epithelium and periglomerular cells (cf. Ashwell, 2012). Mitral cells exhibited moderate labeling of their somata and axons that build the olfactory tract and transmit signals to higher brain regions. In the subependymal layer, deep short axon cells exhibited strong Cygb-IR and colocalized nNOS-immunofluorescence. The external plexiform layer showed few labeled interneurons.

Neurons of the visual pathways (cf. Watson, 2012) as well seem to employ Cygb, which is expressed in a subset of retinal ganglion cells (Schmidt et al., 2005; Ostojić et al., 2006), and seen in the optic chiasm to continue in the optic tract in the present study. Moderately labeled neuronal somata were seen in the (LGB; fourth neuron of the image-forming visual pathway), but the axons of these cells did not appear to be labeled since their termination in the primary visual cortex was not obvious. While zonal and superficial gray layers of the (SC; a relay station parallel to the LGB) were unstained, Cygb-IR fibers in the retino-recipient OP most probably stem from retinal ganglion cells. Faintly labeled neurons were also observed in the deep gray layer of the SC. The presently observed IR fibers in the ventral SCN, supraoptic and paraventricular nuclei may belong to the retino-hypothalamic tract conveying day-night-information to the circadian clock system (cf. Reuss, 2003).

We did not study the cochlear spiral ganglion for Cygb-IR structures yet, but we observed moderately immunofluorescent neurons in downstream auditory sites (cf. Malmierca and Ryugo, 2012), e.g., in the medial geniculate body and SOC of the brainstem. The latter group remains to be tested whether they include olivocochlear neurons, i.e., those that provide the efferent innervation of cochlear hair cells. This will be of particular interest since a parallel study showed that, among several other structures involved in hearing, the auditory efferent neurons of the brainstem contain a distantly related respiratory protein, neuroglobin (Reuss et al., 2016).

In the brainstem, the lateral vestibular nuclei and the magnocellular parts of the medial vestibular nuclei exhibited intensely fluorescent neuronal somata. Neurons in these nuclei receive input predominantly from vestibular end organs and cerebellum, are involved in vestibulo-ocular, -spinal and -cerebellar pathways, and are concerned with the maintenance of head and body posture (cf. Lim and Brichta, 2012). Whether these Cygb-neurons also include efferent neurons of the vestibular nerve must be determined by combined retrograde tracing and immunohistochemistry. The parvocellular region of the medial vestibular nucleus included moderately labeled neurons. The latter were previously described by Hundahl et al. (2010). This region also showed IR puncta in high density probably representing terminals from canal afferents or from spinal cord, cerebellum, or CCo (cf. Lim and Brichta, 2012; Vidal et al., 2015).

### Afferent Innervation of Neurons

An interesting feature that was not considered previously is the presence of the above-mentioned immunofluorescent dots. They most probably represent *afferent* innervation of neurons or neuronal groups, and were observed in addition to the neuron-*efferent* fiber tracts discussed above. In distinct regions, IR puncta that may represent terminals were found in close vicinity of unlabeled neuronal somata. This was seen, e.g., in the AI cortex (Figure 1B) where fibers from subcortical and other cortical regions terminate, and in the caudal IPR (Figure 8A), where IR puncta in high density may represent terminations of axons forming the fasciculus retroflexus originating in the habenular nuclei.

These dots were also evident in the cerebellar cortex, close to (Cygb-negative) Purkinje cell somata. They were seen in low density in the granular layer and in high density in the molecular layer (Figures 8C–F) where Purkinje-cell dendritic trees are present (cf. Bagnall et al., 2013). They were not observed in deep cerebellar nuclei where Purkinje cell axons synapse. It thus appears that only few cerebellar neurons express Cygb, that consequentially cerebellar efferents are Cygb-negative, while the afferents originating in inferior olivary nucleus, spinal cord and brainstem vestibular nuclei contain Cygb.

### Neuroendocrine Structures

Interestingly, both unpaired endocrine glands of the brain (pineal and pituitary) did not exhibit IR structures. In the pineal gland, neither pinealocytes (paraneuronal parenchymal cells secreting serotonin, melatonin and a variety of related and unrelated compounds) nor the afferent fibers originating predominantly in sympathetic superior cervical ganglia (SCG; see Reuss, 2003) for review) seem to express Cygb. The latter is in line with the absence of Cygb-immunoreactivity of SCG ganglion cells, and to the fact that neurons of the MHb (providing additional pineal innervation; cf. Reuss (2003) did not exhibit Cygb-IR processes.

In the pituitary gland, neither cell type in its anterior part was labeled by the Cygb-antibody. These cells produce and secrete a variety of hormones including glandotrophins under the regulation of hypothalamic factors. In the posterior pituitary, also glial pituicytes were not immunofluorescent. The same holds for neuronal fibers, i.e., axons of paraventricular and supraoptic hypothalamic neurons. These neuronal somata were found in the present study to be rather faintly stained and not to exhibit Cygb-IR processes, but it appeared that they receive Cygb-positive input that may originate from retinal, hypothalamic or extra-hypothalamic sites.

### Comparison of Cytoglobin and Neuroglobin Expression

Colocalization of both globins in single neurons was so far only described by Hundahl et al. (2010) in mouse brain. The authors reported double-labeling in about two-thirds of the neurons only in the MHb, pedunculopontine and laterodorsal tegmental nuclei and the locus ceruleus, as well as sporadic coexpression in single neurons in some brain regions. Although we did not conduct double-immunofluorescent detection of both globins, evidence for a considerable overlap (but also for distinct differences) in the expression of Cygb and Ngb results from comparison of Cygb-data as presented here and our rodent Ngb-data as presented previously (Reuss et al., 2002, 2016; Wystub et al., 2003; Hankeln et al., 2004; Laufs et al., 2004; Mitz et al., 2009; Avivi et al., 2010; Schneuer et al., 2012). In all of these cases, it is unknown whether the same cells were labeled with either antibody. Examples are given in the following.

Weak to moderate immunofluorescence, relatively similar for Cygb and Ngb, were seen in some brain regions, e.g., the hypothalamus, thalamus, facial nucleus, cerebellar nuclei (Wystub et al., 2003), and in the SOC (Reuss et al., 2016). Relatively strong expression of Cygb and Ngb was found in the hippocampal pyramidal cell layer; however, their long processes were present only with Cygb-immunofluorescence while they were unseen in the Ngb-labeled material (Hankeln et al., 2004; Laufs et al., 2004).

In the spinal cord, neurons of the gray matter exhibited similarly uniform Cygb- and Ngb-labeling, while those of the functionally connected dorsal root ganglion were Cygb-negative but Ngb-positive. This resulted in a dense network of afferent Ngb-fibers and terminals in the dorsal horn (Hankeln et al., 2004) but not to analogous staining in the Cygb-labeling (this study). Furthermore, cerebellar Purkinje-cells were Cygb-negative but Ngb-positive (Reuss et al., 2002; Wystub et al., 2003). Taken together, it appears that some neurons of the central nervous system express both, Cygb and Ngb, while most neurons express only either of them (in accord to Hundahl et al. (2010).

Another interesting item is that Ngb, originally thought to be expressed only in neurons, is present in glial cells under challenging conditions such as diving (Mitz et al., 2009; Schneuer et al., 2012) or subterranean habitat (Avivi et al., 2010). A similar incidence was yet not observed for Cygb.

### Colocalization of Cytoglobin and Neuronal NO-Synthase

Our double-incubation experiments did not reveal neuronal colocalization of Cygb with the catecholamine synthesis enzyme, TH, in the mouse brain. Both substances, however, were observed in different neuronal structures in the same brain section.

In contrast, the data presented here demonstrate substantial colocalization of Cygb and nNOS in various brain regions. We studied sections from all parts of the mouse brain, however, without conducting a systematical investigation as presented here for the distribution of Cygb.

In many regions (e.g., striatum, CCo, medial vestibular nuclei), we found a one-to-one-colocalization of both proteins in neuronal somata (typical examples given in Figure 9). In other cell groups (e.g., periglomerular neurons in the olfactory bulb), it appeared that cells exhibiting either fluorescence were not always identical. There was clear evidence from all cells that nNOS-immunolabeling was not present in the cell’s nucleus, while Cygb was detected in both, cytoplasm and nucleus.

Only partial colocalization of nNOS in Cygb-positive neurons was observed in the mouse hippocampus presently (data not shown), coinciding with the presence and substantial colocalization of Cygb and nNOS in the hippocampus and in other regions of the rat and mouse brain (Hundahl et al., 2010, 2013).

### Implications for Cygb Function

Although considerable knowledge about the expression patterns and physico-chemical properties of Cygb has been gained in the last 15 years, its actual role(s) in neuronal function remain enigmatic (Burmester et al., 2002; Schmidt et al., 2004; Burmester and Hankeln, 2014). The widespread but distinct distribution of this nerve globin makes it a candidate protein to support particular neuronal functions. It was thus initially tempting to speculate on a supportive role of this globin in oxygen supply, possibly during situations of extended demand. The results of hypoxia studies, however, were conflicting.

On one hand, there was no evidence for a positive correlation of Cygb-expression with oxygen consumption (Hankeln et al., 2005; Schmidt et al., 2005), and no effect of hypoxia or of deep hypothermic circulatory arrest on cerebral Cygb expression in rats (Li et al., 2006) or piglets (Schubert et al., 2010). On the other hand, Cygb-expression was upregulated *in vitro* and *in vivo* in response to hypoxia (Fordel et al., 2004; Schmidt et al., 2004). Recently, it was reported that transgenic overexpression of Cygb reduced hypoxia-induced brain injury (Tian et al., 2013). These studies, however, were conducted using brain homogenates, leaving open whether and how individual Cygb-neurons within a given region may respond to challenging situations.

Previously, an intriguing but yet unproven idea for Cygb function (i.e., by providing O_2_ for collagen maturation) was indirectly supported by the finding that the protein is expressed in active fibroblasts and related cell types that synthesize and secrete the intercellular matrix protein collagen, while inactive fibrocytes, chondrocytes and osteocytes that do not synthesize collagen show reduced levels of Cygb (Schmidt et al., 2004). Further, overexpression of Cygb enhanced the expression of collagen α1(I)-mRNA (Nakatani et al., 2004). There is, however, no evidence for collagen synthesis in neurons. The observation of very different levels of Cygb-immunostaining of neurons in the same brain section and the presence of cytoplasmic *and* nuclear staining in neurons, furthermore, support multiple roles for the globin, depending on the site of expression.

An alternative view is that Cygb has an anti-oxidative property and protects cells from harmful ROS, as suggested by several *in vitro*-studies (Fordel et al., 2007; Hodges et al., 2008; Nishi et al., 2011; see also Raida et al., 2012) for discussion). We would, however, miss a satisfactory explanation why such a generic function should be limited to the described neuronal subpopulations.

Notably, the frequent incidences of colocalization of Cygb and nNOS in brain cells, as seen in the present study and reported previously (Avivi et al., 2010; Hundahl et al., 2010) argue in favor of a functional relation of these proteins. In line with this, the cerebellar cortex output neurons, Purkinje-cells, do not express neuronal NO-synthase (Rodrigo et al., 1994) and are also immunonegative for Cygb (this study). We, therefore, hypothesize that Cygb may provide molecular oxygen which is needed for enzymatic production of NO from L-arginine by nNOS (Leone et al., 1991). Alternatively, Cygb may be instrumental in regulating NO-consumption, as recently suggested for cells of the vasculature (Liu et al., 2013), thereby ensuring NO homeostasis in neurons.

Further open questions are related to Cygb’s presence in the nucleus of neurons (but not of fibroblast-related cells), as the globin itself does not contain a nuclear localization signal. Is there a neuron-specific factor or condition that enables the translocation of Cygb into the nucleus or its retention there after diffusion into the nuclear compartment? Does nuclear Cygb function as a component of chromatin- or gene-regulatory pathways, or does it protect the genetic material from harm? These and other questions warrant further studies on the properties of neuronal cytoglobin.

## Author Contributions

SR conception of the work, acquision and analysis of data, interpretation of data, drafting and revision of manuscript. SW conception of the work, acquision and analysis of data, interpretation of data. UD-K conception of the work, acquision and analysis of data, interpretation of data. TH interpretation of data, revision of manuscript. TB interpretation of data, revision of manuscript. All authors approved the final version of the version to be published. They agree to be accountable for all aspects of the work in ensuring that questions related to the accuracy or integrity of any part of the work are appropriately investigated and resolved.

## Funding

Authors were supported by the Deutsche Forschungsgemeinschaft (Bu956/5 and Ha2103/3), the European Union (QLRT-2001-01548) and the Röttger-Stiftung and Hoffmann-Klose-Stiftung.

## Conflict of Interest Statement

The authors declare that the research was conducted in the absence of any commercial or financial relationships that could be construed as a potential conflict of interest.

## Abbreviations

CA1-3: hippocampal fields
Cygb: cytoglobin
DAPI4: 4′-6-diamidino-2-phenylindole
EFI: extended focal imaging
IR: immunoreactive
MIA: multiple image alignment
MVeMC: medial vestibular nucleus, magnocellular part
MVePC: medial vestibular nucleus, parvocellular part
Ngb: neuroglobin
NO: nitric oxide
nNOS: neuronal nitric oxide-synthase
PBS: phosphate-buffered saline
RT: room-temperature.
